# Incentivising cooperation by judging a group’s performance by its weakest member in neuroevolution and reinforcement learning

**DOI:** 10.3389/frobt.2025.1599676

**Published:** 2025-07-25

**Authors:** Jory Schossau, Bamshad Shirmohammadi, Arend Hintze

**Affiliations:** ^1^BEACON Center for the Study of Evolution in Action, Michigan State University, East Lansing, MI, United States; ^2^Department of MicroData Analytics, Dalarna University, Falun, Dalarna, Sweden

**Keywords:** cooperation, reinforcement learning, neuroevolution, inclusive fitness, group-level selection, fairness, reward schemes

## Abstract

**Introduction:**

Autonomous agents increasingly interact within social domains such as customer service, transportation, and healthcare, often acting collectively on behalf of humans. In many of these scenarios, individually greedy strategies can diminish overall performance, exemplified by phenomena such as stop-and-go traffic congestion or network service disruptions due to competing interests. Thus, there is a growing need to develop decision-making strategies for autonomous agents that balance individual efficiency with group equitability.

**Methods:**

We propose a straightforward approach for rewarding groups of autonomous agents within evolutionary and reinforcement learning frameworks based explicitly on the performance of the weakest member of the group. Rather than optimizing each agent's individual rewards independently, we align incentives by using a “weakest-link” metric, thereby encouraging collective strategies that support equitable outcomes.

**Results:**

Our results demonstrate that this weakest-member reward system effectively promotes equitable behavior among autonomous agents. Agents evolve or learn to balance collective benefit with individual performance, resulting in fairer outcomes for the entire group. Notably, the introduced approach improves overall efficiency, as equitably-minded agents collectively achieve greater stability and higher individual outcomes than agents pursuing purely selfish strategies.

**Discussion:**

This methodology aligns closely with biological mechanisms observed in nature, specifically group-level selection and inclusive fitness theory. By tying the evolutionary and learning objectives to the group’s weakest member, we mimic natural processes that favor cooperative and equitable behaviors. Our findings highlight the importance of incentive structures that consider the collective well-being to optimize both group fairness and individual agent success. Future research should explore how this reward framework generalizes across broader domains and more complex agent interactions.

## 1 Introduction

We are increasingly surrounding ourselves with artificial intelligence-controlled (AI) autonomous systems such as self-driving cars. These systems must be trained by some mechanism, which is usually reinforcement (Watkins and Dayan, 1992), deep reinforcement learning (Sutton and Barto, 2018), or sometimes neuroevolution (Stanley et al., 2019). Regardless of the optimization method, performance must be described by an objective function. While real-world systems like self-driving cars serve as intuitive examples of multi-agent scenarios where fairness and coordination matter, our aim is not to claim immediate applicability of our method to such domains. Rather, we use these analogies to motivate the underlying problem: how can we design objective functions that promote cooperative and equitable behavior in multi-agent systems?

The exploration of such objective functions is not new. The field of reinforcement learning has a rich history of grappling with how to effectively incentivise desired behaviors in complex situations. Specifically, multi-agent reinforcement learning (MARL) has emerged as the area of study dedicated to understanding and engineering cooperation among autonomous entities. To fully appreciate the significance of our proposed minimum reward scheme, it is essential to understand the existing landscape of MARL contributions aimed at promoting cooperative behavior. The following section provides an overview of recent contributions, categorizing approaches based on how they facilitate information sharing and coordinate agent interactions through the design of the objective function.

### 1.1 Background: reinforcement learning for incentivising cooperation

We organize the recent multi-agent reinforcement learning contributions into several broad categories based on how and which information is shared between the agents through the design of the objective function: Incentive Design and Credit Assignment, Coordination and Communication Mechanisms, and Robustness and Adaptability.

Incentive Design and Credit Assignment methods focus on addressing the credit assignment problem. This includes approaches like Intrinsic and Peer Incentives (Zhang et al., 2023), where agents directly reward or punish peers based on their causal influence, and Skill Discovery and Assignment (Li et al., 2024), which uses intrinsic rewards to encourage agents to learn diverse, cooperative skills.

Coordination and Communication Mechanisms methods facilitate effective interaction and information sharing between agents during the expression of their policies. This category includes Mutual Help and Action Expectation (Qiu et al., 2023), where agents share anticipated actions for selective imitation; Consensus and Shared Cognition (Xu et al., 2022), which leverages inferred consensus signals for decentralized cooperation; Communication and Attention Mechanisms (Pu et al., 2022), utilising attention-based networks for broader and more complex communication; and Group-Based Cooperation (Liu et al., 2023), where agents are dynamically grouped using Graph Neural Networks (GNNs) for more structured communication and value decomposition. Trajectory Correlation (Wang et al., 2024) uses behavioral similarity to establish collaborative relationships and improve training.

Robustness and Adaptability includes how MARL systems can maintain cooperation in dynamic or imperfect environments. This includes Adaptive Policy Downsizing (Zhao et al., 2024), which handles agent failures by adaptively weighting historical trajectories, and Weighted Exploration-Exploitation (Liu et al., 2020), which balances individual exploration with shared information to accelerate convergence to optimal joint actions in cooperative tasks.

Each of the above multi-agent reinforcement learning contributions attempts to solve an aspect of behavioral convergence to a cooperative problem through the design of the method and selection of information sharing among agents. There are a diversity of proposed methods because optimality is not necessarily well-understood, or the number of subtle situational variations are too great to enumerate. Otherwise, we would not need reinforcement learning at all. Thus, creating an explosion of enumerated criteria is either unrealistic or impossible. In the automated car example, it is difficult to quantitatively encode “be considerate and optimal” into an objective function.

### 1.2 Group-level selection and inclusive fitness

Nature faces a similar problem during the evolutionary optimization of decision-making. Evolution selects on short-time rewards, making it hard for the complex behavior of cooperation to evolve. Thus, different mechanisms have been proposed that could help cooperation to emerge (Nowak, 2006). The most obvious ones are kin selection (Queller, 1992), group selection (Smith, 1964), and inclusive fitness (Hamilton, 1964a; Hamilton, 1964b). Kin selection is a concept that is hard to transfer into the domain of reinforcement learning, and thus, we leave it out of the discussion here. In biology, group-level selection requires all members of the group to replicate together, let alone receive the same payoff. Inclusive fitness stipulates that the performance of one agent is dependent on another agent. Both these mechanisms find a way to award organisms both individually and from the mutual support of other agents. Group-level and inclusive fitness selection improve cooperation and emphasize the success of the group over the success of the individual. Thus, it should be possible to counter the emergence of selfish AI behavior by using group-level selection or inclusive fitness.

Multiple examples of group-level selection exist and have been identified as drivers of major transitions in evolution, such as the transition from single-to multi-cellular organisms (Rose, 2020) or social insects (Wilson, 1968). While driving cooperation, group-level selection often favors efficient division of labor. Examples include the specialization of soma and germline in multicellularity, or the specialization of queens and workers in social insects. Even in less strict situations where groups of organisms are working together in a synergistic fashion to receive higher benefits, the rewards may not be distributed equally (Owens and Owens, 1978). This division of labor from group-level selection creates a situation where members receive more than they would alone, but still encounter unequal rewards (Bongard, 2000a); Potter et al. (2001) — typically described as a despotic distribution (Andren, 1990). A simple description of despotic distribution is the distribution of benefit over individuals that arises when a set of resources of unique varying benefit are distributed among a population (without sharing, due to contention). The benefit conferred to each individual can be monotonically sorted. The slope, or severity, of this sorted distribution, is what we call the despotic index. A flat slope would indicate a very even and fair distribution of resources. A steep distribution would indicate that one population member has better resources than all others, and the next better than all the remaining, so on down the line. The steepness of this distribution represents the exaggeration of inequality.

Interestingly, the fitness function for optimization in a genetic algorithm can also be applied for optimization in reinforcement learning (Bloembergen et al., 2015). The concept of group-level selection and inclusive fitness can thus be transferred to reinforcement learning. However, inheritance only plays a role in the context of a genetic algorithm wherein populations of agents compete and the fitter ones replicate proportionally more often. This is different from reinforcement learning wherein agents do not replicate. Instead of optimizing a single agent, a group of agents can be trained using an objective function rewarding group performance. This is either accomplished with one policy controlling the actions of all agents at the same time (Buşoniu et al., 2010), or by training independent agents that share information or experiences (Tan, 1993). Thus, inclusive fitness is more akin to using independent policies, while group-level selection is closer to the optimization of a single policy.

Group performance for foraging agents can be assessed by taking the summation of the individually collected rewards or by taking the maximum of those rewards. This typically leads to poor overall performance, diverse behavior, and a higher despotic index (Balch, 1999). Also, the distributed nature of learning poses problems to exploration and learning schedules (Hu and Wellman, 2003). On the other hand, this heterogeneous outcome might be desired to solve other tasks (Panait and Luke, 2005). It has also been argued that global reward schemes do not scale to larger groups and that using individual reward schemes remedies this problem (Wolpert and Tumer, 2002).

This credit assignment difficulty can be summarized as an act of finding the balance between local individual reward that can cause counterproductive interference, and maximizing global group reward that can lead to self-sacrificial inefficiencies at the local scale. For this reason many adaptive methods under the name “shaped reinforcement learning” have been proposed (Matarić, 1997; Bongard, 2000b; Wang et al., 2020), being only a few.

It seems that the literature suggests that a global reward scheme evaluating the maximal or joint effort of a group neither leads to optimal performance, nor does it flatten the despotic index.

Regardless, here we show that assessing the performance of a group by its weakest member leads to optimal performance, while also resulting in a fair distribution of labor and reward–a flat despotic index. We show this for both genetic algorithms and reinforcement learning. The task used here is a foraging task, and performance is optimized by either a genetic algorithm or by reinforcement learning of policies controlling groups of individuals or the entire group. Three different rewarding schemes are compared.

• MEAN: the resources are pooled and then fairly distributed among the four agents

• MINIMUM: each agent gets the same score defined by the agent who collected the least food

• MAXIMUM: each agent gets the same score defined by the agent who collected the most food (a control)

We will show that the MINIMUM reward scheme indeed leads to high performance while also satisfying a low despotic index.

## 2 Methods

### 2.1 The foraging task

The foraging task requires four agents to collect food at the same time, which requires no explicit cooperation. After the foraging period is over, the amount of food each agent has collected is evaluated and used to determine either fitness for the Genetic Algorithm (GA) or rewards for RL. However, when training a policy using RL, the state and action space must be kept small for feasible runtimes, so we use a simplified version.

We chose the foraging task because it offers a clear, interpretable environment where both cooperation and competition can emerge naturally. Its simplicity allows us to isolate the effect of reward schemes without introducing confounds from task complexity. The fixed team size of four agents ensures consistent interaction dynamics, while the spatial layout ( 16×16 grid) provides ample opportunity for agents to interfere with, support, or ignore each other. Each agent is equipped with basic sensors (e.g., detecting objects ahead and hearing other agents’ beeping signals) and actuators (e.g., movement, giving food), allowing for both implicit and explicit forms of coordination. Rewards are determined at the end of each episode by evaluating the amount of food each agent has collected. Depending on the reward scheme (MINIMUM, MEAN, or MAXIMUM), this group-level outcome is then uniformly assigned to all group members. The inclusion of a give-food action and a limited communication channel via beeps enables coordination strategies that directly reflect the constraints imposed by the reward function, thereby making this task particularly suitable for evaluating the fairness and effectiveness of cooperative incentives.

#### 2.1.1 Environment using a genetic algorithm

Four agents are placed in the corners of a rectangular room ( 16×16 tiles). The room is filled with tokens for the agents to collect—for analogy we can think of these tokens as food. The agents can move forward onto food and automatically collect it, they can turn left or right, do nothing, and also utter a far-ranging beeping signal as a form of communication. Agents can further deposit already-collected food in front of them, or if an agent is directly in front then the food is transferred to that agent. Agents receive inputs of how much food they collected, what is immediately in front of them, and the beeping signals of other agents.

##### 2.1.1.1 Objective function for the genetic algorithm

The four agents to forage in the environment are either chosen randomly from the population (without replacement) and evaluated once, or each agent in the population is cloned three times to create a group of four identical agents that are evaluated in the environment. Agents are allowed to roam the environment and collect food for 100 time points (updates). The amount of food each agent has at the end is recorded and the fitness functions depend on the amount of food each agent i collected $F_{i}$ . Regardless of individual or clonal groups, the performance of the group (W) is based on the individually collected amount of food $\left(F_{i}\right)$ and is determined using one of three statistical methods: MEAN (see Equation 1, MAXIMUM (Equation 2), and MINIMUM Equation 3).

$$W_{\mathrm{MEAN}}=\frac{1}{4}\overset{i<4}{\underset{i=1}{\sum }}F_{i}$$ (1)

$$W_{\mathrm{MAX}}=\max\left(F_{0},F_{1},F_{2},F_{3}\right)$$ (2)

$$W_{\mathrm{MIN}}=\min\left(F_{0},F_{1},F_{2},F_{3}\right)$$ (3)

##### 2.1.1.2 Markov brain neural networks

Agents are controlled by Markov Brains (MB) (Hintze et al., 2017). MBs are neural networks that replace the commonly used aggregation and threshold functions with probabilistic or deterministic logic gates as well as mathematical operators to compute the state of nodes. The exact connectivity and use of computational units is defined by a genome subject to point mutations, deletions, and gene duplications. These mutations allow the computational units to connect to seven sensor nodes, three actuators, and nine hidden nodes. Two of the five sensor nodes convey what lies in front of the agent, and three for the beeping of other agents. The two output nodes encode the movement of the agent (left, right, nothing, and moving forward/eating/giving food). The nine hidden nodes can store information in a recurrent fashion, and thus allow memory.

##### 2.1.1.3 Parameters for the genetic algorithm

We randomly generate 100 agents at the start of the experiment and depending on the chosen method we evaluate their fitness every generation using only inclusive fitness or group-level selection. To evaluate inclusive fitness, we randomly select four agents with replacement and evaluate them as a group. This process is repeated four times per generation to ensure thorough evaluation. For group-level selection, we clone each agent three times and evaluate the four identical agents together. We then remove three of them, so that the group’s performance only affects the replication of one agent. Optimization proceeds using roulette wheel selection over 50,000 generations.

#### 2.1.2 Environment using Q-learning

The environment for Q-learning is smaller (8×8) and agents can see the entire area. Empty tiles are encoded as 0, food is 1, other agents are −1 , and the agent to be controlled is represented as −0.5 . Thus, the state of the environment s is a tuple of length 64. These continuous values are chosen to also allow for the deep-Q learning variation. Agents in this environment move in four cardinal directions (North, South, East, West). When an agent tries to move to a tile occupied by another agent, then one piece of food is transferred from the moving agent to the stationary agent. Because all agents see each other, then no further communication is necessary.

The reason for doing this is to allow Q-learning. In Q-learning, the policy maps each possible environment state to an expected reward for each possible action. So, the environment state must be clear and unambiguous. The GA optimization environment is bigger and has the same states as the Q-learning environment, but agents only see the environment from their own viewpoint. This can create ambiguous states for Q-learning. For example, if all agents see only food in front of them, then this does not tell us whether other locations are empty or full, which is important for Q-learning. The mechanisms and rewards are the same for both the GA and Q-learning environments, but in the GA environment agents have limited information, while in the Q-learning environment agents have perfect information.

##### 2.1.2.1 Rewards for reinforcement learning

We distinguish between two types of machine learning policy: decentralized and centralized. With decentralized policies, each agent has its own Q-matrix, which is reinforced independently from other agents. The Q-matrix estimates the rewards for movement of that agent in each of the four directions. In centralized policies, a single policy controls all four agents and estimates rewards for all possible combinations of their movements. With four agents each able to move independently in four directions, the centralized policy estimates rewards for 256 $\left(4^{4}\right)$ possible actions.

##### 2.1.2.2 Q-learning

Q-learning proceeded by reinforcing actions for agents foraging for food in a food-saturated environment. The foraging process consisted of 50 steps for each epoch. Each agent’s action was based on its respective Q-matrix predictions. We implemented a greedy-epsilon decay exploration strategy with an ϵ probability initialized at 1.0 and decaying at a rate of 0.999. A random action was selected during exploration steps. After each step, we recorded the rewards, actions, and states for later experience replay. Rewards were defined according to the three objective functions (MEAN, MAXIMUM, and MINIMUM) outlined above. Agents were trained using experience replay, selecting 2,000 experiences randomly from a buffer with a maximum length of 50,000. We used a deque to record experiences such that newer experiences replaced older ones.

Q-learning is a stochastic approximation to the Bellman optimality equation, in which the agent incrementally estimates action values based on sampled transitions from its experience. We therefore calculated new rewards using a variant of the Bellman equation (Equation 4):

$$Q^{\mathrm{new}}\left(s_{t},a_{t}\right)\leftarrow Q\left(s_{t},a_{t}\right)+\alpha \left(r_{t}+\gamma \underset{a}{\max}Q\left(s_{t+1},a\right)-Q\left(s_{t},a_{t}\right)\right),$$ (4)

which uses a learning rate α of 0.8 (Equation 5):

$$Q^{\mathrm{new}}\left(s_{t},a_{t}\right)\leftarrow \left(1.0-\alpha \right)Q\left(s_{t},a_{t}\right)+\alpha \left(r_{t}+\gamma \underset{a}{\max}Q\left(s_{t+1},a\right)\right)$$ (5)

Optimization was performed for 10,000 epochs, and policies stopped improving after an average of 5,000 generations. We repeated each stochastic experiment 40 times.

##### 2.1.2.3 Q-matrix pruning

The environment consists of 8×8 tiles, which results in $4^{64}$ possible states for the agents’ positions. Each tile can have one of four possible states: empty, food, other, or self. This implies a dense Q-matrix of $4^{64}\times 256$ for the group policy condition. Unfortunately, we did not have sufficient fast memory storage available during the research. To overcome this issue, we created a sparse Q-matrix using a dictionary, which is similar to the method used in (Nishio and Yamane, 2018). When the policy is consulted, a query is made to the dictionary for a reward vector based on the hash of an integer representation of the state. If a state does not exist in the dictionary, a random reward vector is created for that state. All states are tagged with a timestamp of the last accessed epoch. Training an agent’s policy over 50,000 steps would still overflow the memory of a conventional computer, so we pruned the dictionary of entries that were unused for at least 2,000 epochs.

Pruning a policy reduces its memory footprint, but may also detrimentally affect learning. When a state is not visited, then it has a uniform estimated reward distribution over all possible actions, while every visited state adapts to the expected reward distribution due to reinforcement. Removing a previously visited state removes those adaptations and effectively “pretends” as if the state has never been reached before. We could imagine other contexts wherein this pruning could mitigate learning. However, in this study, a non-rewarding action leads an agent to an empty tile, while a rewarding action leads an agent to one with food. Thus, the learning landscape is not too deceptive, and the expected rewards stored in the policy will lead an agent away from empty tiles and toward food. Therefore, it is assumed that low-rewarding states will be abandoned quickly by the policy. This pruning will erase the experience of certain rewards, likely slowing learning. However, these states remain unrewarding, so they will again be abandoned quickly by the policy.

Similarly, the exploration probability ensures that agents visit formerly unvisited states to possibly discover higher rewards. This exploration probability is conventionally annealed over the course of learning, allowing the policy to converge on a solution to a static problem. This progression from exploration to exploitation reduces the space of possible states visited by the agent, and consequently a smaller part of the policy is needed. Pruning non-visited states in a converged policy will not affect the actions of agents or the rewards they collect. Here, we prune states when they have not been visited for for 2,000 epochs. If this pruning threshold is very low, then learning would be seriously limited. If the pruning threshold is very high, then it would have no effect on learning but result in a large memory footprint. Because we do find proper solutions, then we assume that the chosen threshold is a good compromise between meeting memory constraints and not seriously compromising policy convergence.

This pruning method also assumes consistent starting locations. As such, the exploration of the policy can be imagined as a search tree, where learning will quickly avoid unrewarding choices and seek out rewarding ones. It will not cause harm to prune these unrewarding branches. If using arbitrary starting positions, then the analogy to the search tree falls apart and time until safe pruning may be prohibitively high.

In summary, a sparse and pruned policy achieves memory efficiency while allowing reinforcement convergence, and it remains an open question if this scheme could be applied to other situations.

Policies can be prohibitively large in some Reinforcement Learning architectures. Deep Q-learning (DQN) was developed to counter this problem. In DQN the policy does not discretely estimate every possible reward and state-action pair, but estimates the mathematical relationship between rewards and state-action pairs based on experience using a deep neural network. While DQN is a powerful method, it couples the learning efficiency of Q-learning to the learning efficiency of deep neural network backpropagation. Backpropagation potentially obfuscates results for this experiment and so it was not used here.

##### 2.1.2.4 Statistical comparisons

We examined the statistical significance between conditions using the two-sample Kolmogorov–Smirnov (KS) test. The KS test was chosen because it is a non-parametric method that compares the full distribution of two independent samples without assuming normality, making it well-suited for our performance data, which may be non-Gaussian or multimodal. We compared all experimental conditions against the GroupLS-MEAN and GroupLS-MINIMUM baselines, resulting in m=10 pairwise tests. Bonferroni correction was applied to control for multiple hypothesis testing with an adjusted significance threshold of $p<\frac{0.01}{m}$ .

## 3 Results

### 3.1 Optimization by genetic algorithm

**FIGURE 1:**
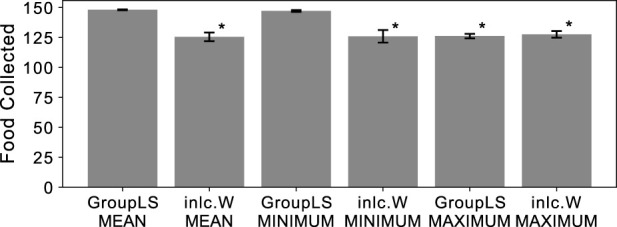
Average food collected by the entire group of agents that were optimized by a genetic algorithm, for different reward and selection regimes. Group-level selection (GroupLS) uses clonal groups whereas Inclusive Fitness (Incl.W) uses individual selection. Error bars indicate 95% confidence intervals over the 40 replicates performed. An asterisk (∗) indicates statistical significance of a 2-sample Kolmogorov-Smirnov test ( $p<\frac{0.01}{10}$ , Bonferroni corrected) when comparing to both GroupLS-MEAN and GroupLS-MINIMUM, which were not significantly different from each other.

Populations with groups can either experience group-level selection during agent group reproduction, or experience inclusive fitness effects through independent reproduction. To distinguish between both processes in our experiments we created groups for selection either from random population members (inclusive fitness effects) or clones of a single member (group-level selection). We determined which group-selection method and reward scheme combination leads to the highest performance of a group for the reward schemes MEAN, MAXIMUM, and MINIMUM. We find that all three reward schemes generally result in high performance, but that clonal groups using MEAN or MINIMUM reward schemes outperform the others (see Figure 1).

**FIGURE 2:**
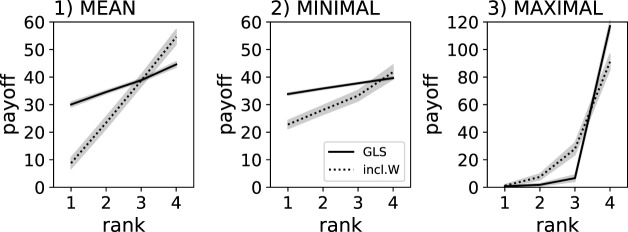
Individual food collected by agents trained using a genetic algorithm. Solid lines indicate group-level selection using clonal groups (GLS), dotted lines indicate groups composed of genetically independent individuals experiencing only inclusive fitness effects (inlc.W). Individual results are ranked (x-axis), which is a sorting by the amount of food collected relative to the other agents in the group. The gray shadows indicate the 95% confidence intervals from the 40 replicate experiments. Three different reward schemes were compared: MEAN, MINIMUM, and MAXIMUM - note the different y-scale for the MAXIMUM reward scheme, which centralizes all collected food to one agent, while the other regimes ensure that all agents are involved in collecting the food.

**FIGURE 3:**
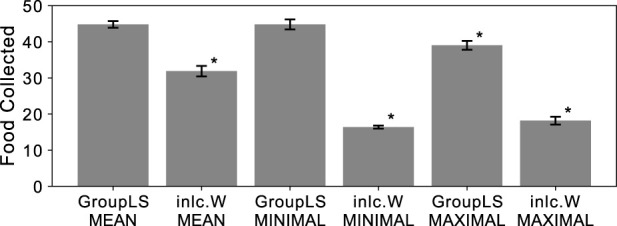
Average food collected by groups of agents optimized using Q-learning. Group-level selection (GroupLS) indicate policies controlling all agents of the group at the same time, while groups of policies controlling only one agent at a time are analogous to inclusive fitness (inlc.W). Error bars indicate 95% confidence intervals over the 40 replicates performed. An asterisk (∗) indicates statistical significance of a 2-sample Kolmogorov-Smirnov test ( $p<\frac{0.01}{10}$ , Bonferroni corrected) when comparing to both GroupLS-MEAN and GroupLS-MINIMUM, which were not significantly different from each other.

We also investigated the effect of replication method and reward scheme on the despotic index. As expected, the despotic index was highest when using the MAXIMUM reward scheme regardless of group-level selection or inclusive fitness (see Figures 2, 3). The next steepest despotic index can be found when using the MEAN reward scheme, with group-level selection leading to a flatter hierarchy than inclusive fitness (see Figure 2). Finally, group-level selection with MINIMUM reward scheme resulted in a nearly equal resource distribution, indicating the most fair behaviors and outcomes for all agents in the group (see Figure 2). Using inclusive fitness and the MINIMUM reward scheme results in a flatter distribution of resources compared to MEAN and MAXIMUM, but is still steeper than group-level selection when using the MINIMUM reward scheme.

These results show that group-level selection using the MEAN and MINIMUM reward schemes achieve comparable group performance, with no statistically significant difference between them. However, the MINIMUM scheme uniquely results in a substantially flatter despotic index, highlighting its strength in promoting equitable workload distribution without compromising efficiency. In contrast, while the MEAN scheme also reduces inequality to some extent, it does not achieve the same level of fairness as the MINIMUM scheme. Under the MAXIMUM reward scheme, group-level selection instead results in a steeper hierarchy, with one agent typically collecting a disproportionately large share of the resources.

### 3.2 Optimization using reinforcement learning

As discussed before, the concepts of group-level selection and inclusive fitness do not perfectly translate to reinforcement learning since neither policies nor agents “replicate.” However, using one policy to control all four agents contemporaneously can be equated with group-level selection—called centralized control (Goldman and Zilberstein, 2003). In centralized control, rewards are used to reinforce all agent behaviors simultaneously. In contrast, inclusive fitness resembles using four independent policies—called decentralized control. Rewards received by one agent only directly affect the policy of that agent, while the actions of other agents are only indirectly included through the reward scheme and lifetime interaction state changes.

In all cases (MEAN, MINIMUM, and MAXIMUM) centralized control outperforms decentralized control, and MEAN and MINIMUM reward schemes yield better-performing agents than MAXIMUM when using centralized control.

**FIGURE 4:**
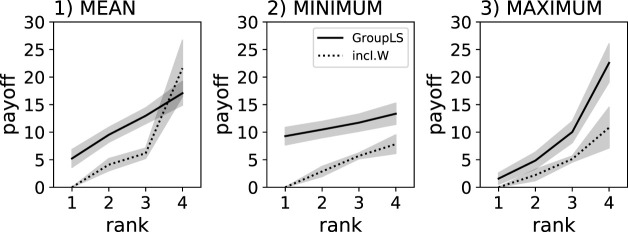
Individual food collected by agents controlled by Q-learning policies. Solid lines show the centralized control group-level selection condition, where a single policy controls the actions of all group members (GroupLS). Dotted lines show groups composed of agents controlled by independent policies (inlc.W). Individual results are ranked (x-axis), which is a sorting by amount of food collected relative to the other agents in the group. The gray shadows indicate the 95% confidence intervals from the 40 replicate experiments. Three different reward schemes were compared: MEAN, MINIMUM, and MAXIMUM.

Notably the despotic index is flattest when using the MINIMUM reward scheme and steepest when using the MAXIMUM reward scheme (see Figure 4). In all cases, using decentralized (group-level) control policies resulted in flatter hierarchies and thus “fairer” agents.

## 4 Discussion

Reinforcement learning and genetic algorithms are powerful tools that can effectively optimize agent behavior toward a predefined goal. However, designing the fitness function to achieve this goal is more an art than a science, especially for genetic algorithms. For instance, there is much research on how to quantify success for multiple objectives—or difficult-to-define objectives—with researchers not only engineering new mathematical algorithms, but drawing inspiration from grasshoppers, ant colonies, and immune systems, to name a few (Mirjalili et al., 2018; Ning et al., 2019; Lin et al., 2018). Being considerate to other members of a group while also maximizing personal reward is one of these difficult cases. Agents incentivised to increase their personal rewards may do so at the expense of other agents, while requiring them to be considerate may hamper their individual success. The challenge lies in finding the right balance between these two extremes.

In this study, we focused on three intuitive reward aggregation schemes: *MINIMUM*, *MEAN*, and *MAXIMUM*. These were selected to represent canonical strategies for cooperative behavior—optimizing for the weakest, the average, and the strongest performer in a group, respectively. Their simplicity allows for transparent interpretation and comparative analysis of fairness and performance trade-offs. However, we acknowledge that other summary statistics may capture different aspects of group dynamics. For example, the *median* could offer robustness to outliers, while the *harmonic mean* or *softmin* might interpolate between the *MINIMUM* and *MEAN* regimes. Exploring such alternatives, including adaptive or learned aggregation functions, is a promising direction for future research and could uncover more nuanced control over cooperative behavior and equity outcomes. We found *MINIMUM* to be a simple reward scheme that increases individual rewards while also promoting “fairness” among agents.

“Fairness” — the minimization of the despotic index—is better achieved through rewarding a group for the performance of its weakest member, not by the average of all members or the success of its strongest member. Group performance under this MINIMUM reward scheme is on par with rewarding the average, and is better than rewarding the best, at least for group-level selection. Similar maximization of a minimum objective in reinforcement learning has been shown to achieve decent performance (Tang et al., 2020). However, the flattening of the despotic index resulting from this minimum objective was unknown. Here we study exactly this distribution effect of selection scheme at the interface of group-level selection and inclusive fitness.

Group-level selection and inclusive fitness are mechanisms known from biological evolution and thus translate easily to genetic algorithms. Biological group-level selection is very similar to reinforcement learning of a single centralized control policy rewarded for group performance. In a biological context, inclusive fitness describes the effect of other agents’ behavior on an individual’s fitness. Using different policies in reinforcement learning for different agents that receive independent rewards while still experiencing the effect of each other’s actions resembles this biological mechanism. However, the exact degree to which these biological processes align with reinforcement methods remains open for debate.

To assess how task-dependent our results are, we applied the same reward schemes to a second task involving agent navigation across an intersection (see Supplementary Material, Section 1). In this task, agents receive delayed, sparse rewards based on how quickly they reach a predefined goal. Despite the structural and strategic differences from the foraging task, the MINIMUM reward scheme again led to a flatter payoff distribution and better group performance, especially under group-level selection. These results suggest that the benefits of the MINIMUM reward scheme are not specific to foraging, but may apply more broadly to cooperative tasks. To better understand the generality and practical utility of the MINIMUM reward scheme, future studies should apply it to richer cooperative contexts, including human-agent interactions or groups of large language model (LLM)-driven agents. The MINIMUM, MAXIMUM, and MEAN reward schemes were applied to Q-learning and not to deep Q-learning, which would replace the policy with a neural network to estimate future rewards based on the current state. However, our results should easily generalize to the deep Q-learning domain, as all principles examined here concern action-reward optimization, and not the recollection accuracy of these rewards.

The cooperative forging task used here allows for synergistic behaviors as well as antagonistic inefficient behaviors. For the MAXIMUM reward scheme we found some agents collecting comparatively little food, thereby limiting overall group performance. This can be interpreted as a form of cooperation due to division of labor, where one agent is collecting resources while the others get out of the way. Alternatively, since agents can give resources to other agents, they may actively pool resources in one agent. As such, the MAXIMUM reward scheme cannot be directly compared to the MINIMUM reward scheme because they encourage two entirely different strategies. However, this is exactly the result of this research: Using the MINIMUM reward scheme encourages an increase in every agent’s performance in an equal fashion avoiding an unfair distribution. This flattening of the despotic index is a consequence that pure individual MEAN or MAXIMUM reward schemes do not select for. It would be interesting to test if the MINIMUM reward scheme is equally effective for economic and social structures to create fair but profitable resource distributions.

This simple reward scheme is different from other team-based machine learning reward functions in its simplicity. For instance, one of the most popular recent proposals in this area is OpenAI’s Five Heroes algorithm (Berner et al., 2019). This algorithm uses a teamwork hyperparameter they call “team spirit” that is annealed during training to balance exploration of individual skill and exploitation of team strategy. Mathematically defining this trade-off places the burden of specification in the hands of the experimenter, who must adapt the annealing schedule to each problem domain. If they do not, then the algorithm may completely fail for dynamic problems that fundamentally change at the wrong time during the annealing schedule. The proposed reward scheme here does not have this hyperlimitation.

It is important to distinguish between minimizing a global objective and using the MINIMUM operator as a reward aggregation scheme. In prior work, multi-agent systems have been trained to minimize quantities such as distance to goals or total travel time, but in those cases, the optimization is applied to a scalar group objective. Our use of the MINIMUM scheme refers to aggregating individual agent outcomes by assigning the group’s reward according to the lowest-performing agent. For example, in a task where all agents seek to reach a goal, traditional minimization would reduce the average or total distance across the group, whereas the MINIMUM scheme would evaluate performance solely based on the agent furthest from their goal. This subtle but important difference underlies our focus on fairness and workload equalization, rather than efficiency alone. In practice, using the MINIMUM reward scheme might not even impose significant overhead. Imagine reinforcement training of a self-driving car in a virtual environment also simulating other cars. Using the minimum reward that any of the virtual cars obtains is a simple addition to such an simulation, because all car behaviors are modeled anyway. Whether or not those other cars should be controlled by a centralized or decentralized policy, or if the MINIMUM reward scheme functions the same way in a mix of heterogeneous policies—especially selfish ones—remains an open question for future research.

We proposed a simple and biologically-inspired reward aggregation scheme in which the group’s success is determined by its weakest member. This MINIMUM reward scheme promotes fairness by flattening the distribution of rewards among agents, while maintaining competitive performance across both genetic algorithms and reinforcement learning frameworks. Its simplicity makes it easy to implement even in complex simulations, and it provides a consistent selection or reinforcement signal that encourages equitable contributions. While our findings demonstrate the benefits of this approach in controlled environments, its applicability to more dynamic, heterogeneous, or human-in-the-loop systems remains an open question. Future work should investigate how well the MINIMUM scheme generalizes to real-world multi-agent scenarios.

## Data Availability

The datasets presented in this study can be found in online repositories. The names of the repository/repositories and accession number(s) can be found below: https://github.com/Hintzelab/ https://osf.io/agxze/.

## References

[B1] Andren H. (1990). Despotic distribution, unequal reproductive success, and population regulation in the jay garrulus glandarius l. Ecology 71, 1796–1803. 10.2307/1937587

[B2] Balch T. (1999). “The impact of diversity on performance in multi-robot foraging,” in Proceedings of the third annual conference on Autonomous Agents, 92–99.

[B3] Berner C. Brockman G. Chan B. Cheung V. Debiak P. Dennison C. (2019). Dota 2 with large scale deep reinforcement learning. arXiv Prepr. arXiv:1912.06680. 10.48550/arXiv.1912.06680

[B4] Bloembergen D. Tuyls K. Hennes D. Kaisers M. (2015). Evolutionary dynamics of multi-agent learning: a survey. J. Artif. Intell. Res. 53, 659–697. 10.1613/jair.4818

[B5] Bongard J. C. (2000a). “The legion system: a novel approach to evolving heterogeneity for collective problem solving,” in European conference on genetic programming (Springer), 16–28.

[B6] Bongard J. C. (2000b). “The legion system: a novel approach to evolving heterogeneity for collective problem solving,” in European conference on genetic programming (Springer), 16–28.

[B7] Buşoniu L. Babuška R. De Schutter B. (2010). Multi-agent reinforcement learning: an overview. Innovations multi-agent Syst. applications- 1, 183–221. 10.1007/978-3-642-14435-6_7

[B8] Goldman C. V. Zilberstein S. (2003). “Optimizing information exchange in cooperative multi-agent systems,” in Proceedings of the second international joint conference on Autonomous agents and multiagent systems (Association for Computing Machinery), 137–144. 10.1145/860575.860598

[B41] Hamilton W. D. (1964a). The genetical evolution of social behaviour. I. J. Theor. Biol. 7, 1–16. 10.1016/0022-5193(64)90038-4 5875341

[B9] Hamilton W. D. (1964b). The genetical evolution of social behaviour. II. J. Theor. Biol. 7, 1–52. 10.1016/0022-5193(64)90039-6 5875341

[B10] Hintze A. Edlund J. A. Olson R. S. Knoester D. B. Schossau J. Albantakis L. (2017). Markov brains: a technical introduction. arXiv Prepr. arXiv:1709.05601. 10.48550/arXiv.1709.05601

[B11] Hu J. Wellman M. P. (2003). Nash Q-learning for general-sum stochastic games. J. Mach. Learn. Res. 4, 1039–1069. 10.1162/1532443041827880

[B12] Li T. Bai C. Xu K. Chu C. Zhu P. Wang Z. (2024). Skill matters: dynamic skill learning for multi-agent cooperative reinforcement learning. Neural Netw. official J. Int. Neural Netw. Soc. 181, 106852. 10.1016/j.neunet.2024.106852 39522419

[B13] Lin Q. Ma Y. Chen J. Zhu Q. Coello C. A. C. Wong K.-C. (2018). An adaptive immune-inspired multi-objective algorithm with multiple differential evolution strategies. Inf. Sci. 430, 46–64. 10.1016/j.ins.2017.11.030

[B14] Liu H. Zhang Z. Wang D. (2020). Wrfmr: a multi-agent reinforcement learning method for cooperative tasks. IEEE Access 8, 216320–216331. 10.1109/ACCESS.2020.3040985

[B15] Liu W. Peng L. Wen L. Yang J. Liu Y. (2023). Decomposing shared networks for separate cooperation with multi-agent reinforcement learning. Inf. Sci. 641, 119085. 10.1016/j.ins.2023.119085

[B16] Matarić M. J. (1997). “Reinforcement learning in the multi-robot domain,” in Robot colonies (Springer), 73–83.

[B17] Mirjalili S. Z. Mirjalili S. Saremi S. Faris H. Aljarah I. (2018). Grasshopper optimization algorithm for multi-objective optimization problems. Appl. Intell. 48, 805–820. 10.1007/s10489-017-1019-8

[B18] Ning J. Zhang C. Sun P. Feng Y. (2019). Comparative study of ant colony algorithms for multi-objective optimization. Information 10, 11. 10.3390/info10010011

[B19] Nishio D. Yamane S. (2018). “Faster deep q-learning using neural episodic control,”, 1. IEEE, 486–491. 10.1109/compsac.2018.00075

[B20] Nowak M. A. (2006). Five rules for the evolution of cooperation. science 314, 1560–1563. 10.1126/science.1133755 17158317 PMC3279745

[B21] Owens M. J. Owens D. D. (1978). Feeding ecology and its influence on social organization in brown hyenas (hyaena brunnea, thunberg) of the central kalahari desert. Afr. J. Ecol. 16, 113–135. 10.1111/j.1365-2028.1978.tb00433.x

[B22] Panait L. Luke S. (2005). Cooperative multi-agent learning: the state of the art. Aut. Agents Multi-Agent Syst. 11, 387–434. 10.1007/s10458-005-2631-2

[B23] Potter M. A. Meeden L. A. Schultz A. C. (2001). “Heterogeneity in the coevolved behaviors of mobile robots: the emergence of specialists,” in Proceedings of the 17th International Joint Conference on Artificial Intelligence (IJCAI). (Seattle, WA: Morgan Kaufmann) 17, 1337–1343. 10.5555/1642194.1642273

[B24] Pu Z. Wang H. Liu Z. Yi J. Wu S. (2022). Attention enhanced reinforcement learning for multi agent cooperation. IEEE Trans. Neural Netw. Learn. Syst. 34, 8235–8249. 10.1109/TNNLS.2022.3146858 35180087

[B25] Qiu Y. Jin Y. Yu L. Wang J. Zhang X. (2023). “Promoting cooperation in multi-agent reinforcement learning via mutual help,” in Icassp 2023 - 2023 IEEE international conference on acoustics, speech and signal processing (ICASSP), 1–5doi. 10.1109/ICASSP49357.2023.10095800

[B26] Queller D. C. (1992). A general model for kin selection. Evolution 46, 376–380. 10.1111/j.1558-5646.1992.tb02045.x 28564031

[B27] Rose C. J. (2020). Germ lines and extended selection during the evolutionary transition to multicellularity. J. Exp. Zoology Part B Mol. Dev. Evol. 336, 680–686. 10.1002/jez.b.22985 32681710

[B28] Smith J. M. (1964). Group selection and kin selection. Nature 201, 1145–1147. 10.1038/2011145a0

[B29] Stanley K. O. Clune J. Lehman J. Miikkulainen R. (2019). Designing neural networks through neuroevolution. Nat. Mach. Intell. 1, 24–35. 10.1038/s42256-018-0006-z

[B30] Sutton R. S. Barto A. G. (2018). Reinforcement learning: an introduction. MIT press.

[B31] Tan M. (1993). “Multi-agent reinforcement learning: independent vs. cooperative agents,” in Proceedings of the tenth international conference on machine learning, 330–337.

[B32] Tang J. Song J. Ou J. Luo J. Zhang X. Wong K.-K. (2020). Minimum throughput maximization for multi-uav enabled wpcn: a deep reinforcement learning method. IEEE Access 8, 9124–9132. 10.1109/access.2020.2964042

[B33] Wang J. Zhang Y. Kim T.-K. Gu Y. (2020). Shapley q-value: a local reward approach to solve global reward games. Proc. AAAI Conf. Artif. Intell. 34, 7285–7292. 10.1609/aaai.v34i05.6220

[B34] Wang S. Du H. Zhou Y. Zhao Z. Zhang R. Chen W. (2024). Enhancing collaboration in multi-agent reinforcement learning with correlated trajectories. Knowl. Based Syst. 305, 112665. 10.1016/j.knosys.2024.112665

[B35] Watkins C. J. Dayan P. (1992). Q-learning. Mach. Learn. 8, 279–292. 10.1023/a:1022676722315

[B36] Wilson E. O. (1968). The ergonomics of caste in the social insects. Am. Nat. 102, 41–66. 10.1086/282522

[B37] Wolpert D. H. Tumer K. (2002). “Optimal payoff functions for members of collectives,” in Modeling complexity in economic and social systems (World Scientific), 355–369.

[B38] Xu Z. Zhang B. Li D. Zhang Z. Zhou G. Fan G. (2022). Consensus learning for cooperative multi-agent reinforcement learning, 11726–11734.

[B39] Zhang T. Liu Z. Pu Z. Yi J. (2023). Peer incentive reinforcement learning for cooperative multiagent games. IEEE Trans. Games 15, 623–636. 10.1109/TG.2022.3196925

[B40] Zhao Z. Zhang Y. Wang S. Zhang F. Zhang M. Chen W. (2024). Qdap: Downsizing adaptive policy for cooperative multi-agent reinforcement learning. Knowl. Based Syst. 294, 111719. 10.1016/j.knosys.2024.111719

