# Supplementary material for: Incentivising cooperation by judging a group’s performance by its weakest member in neuroevolution and reinforcement learning
Source: Front Robot AI. 2025 Jul 25;12:1599676. doi: 10.3389/frobt.2025.1599676 (PMC12331510; doi:10.3389/frobt.2025.1599676)
Supplement: Supplementary file 1 [file DataSheet1.pdf]

Supplementary Material

1 AGENT NAVIGATION TASK FOR REINFORCEMENT LEARNING

In this simple navigation task, four agents are placed at an intersection of four roads (see Figure S1) and must cross the intersection and proceed to exit the other side. Agents are color coded and should leave the intersection at a specific outlet matching their own color. Agents are randomly placed at an outlet matching one of the other agents, ensuring that all agents must cross the intersection to reach their own matching outlet. Agents can move in all four directions (up, down, right, and left), but cannot pass through one another or leave the road. Once an agent leaves through its designated outlet, it is removed and the simulation continues for the remaining agents for up to 500 updates. Agents cannot leave the environment through a mismatched outlet.

Agents are trained using Q-learning, as described in the main text. The only difference is that individual rewards are determined for each agent by the time it leaves the intersection through its matching outlet. This reward is calculated as $500 - steps$, so that agents leaving the environment are rewarded for their expediency. This environment is interesting because it provides only one opportunity to complete the task, whereas in the foraging task agents are gradually rewarded for their behavior. In addition, agents can more easily block an outlet—thus making it impossible for another agent to succeed. Lastly, agents do not only experience 50 steps but must deal with delayed rewards for up to 500 steps. This delay makes Q-learning much more difficult, rendering this navigation task a harder and more competitive challenge than the foraging environment.

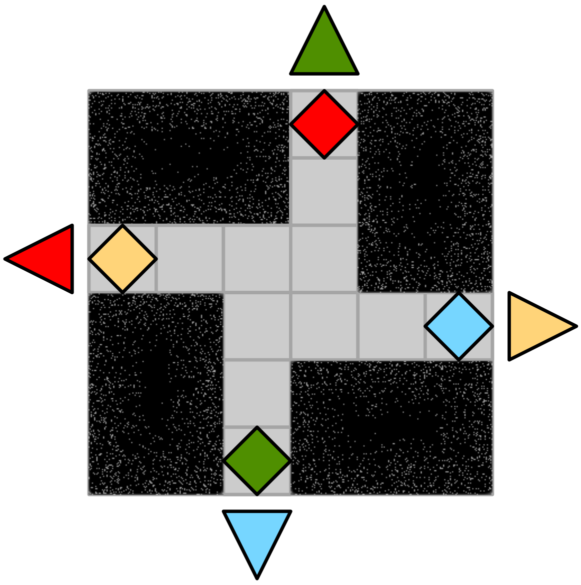

Figure S1. Agent navigation task. A 6×6 area with an intersecting street (gray) and four outlets (color coded arrows, clockwise from the top: green, yellow, blue, and red). Agents (diamonds) must navigate to an outlet matching their color. Outlet positions and colors were kept constant throughout the experiment, while the four agents were randomly placed at the outlets such that they were never adjacent to their own outlet.

Agents were trained using group-level selection or inclusive fitness modes, and all three reward functions (MEAN, MINIMAL, or MAXIMUM) were tested independently 40 times. When comparing the average score (see Figure S2), two observations emerge. First, inclusive fitness fails to solve the task and is outperformed by group-level selection regardless of the reward scheme—likely due to the competitive nature of the environment. Secondly, the MINIMAL reward scheme results in better agent performance compared to MEAN or MAXIMUM.

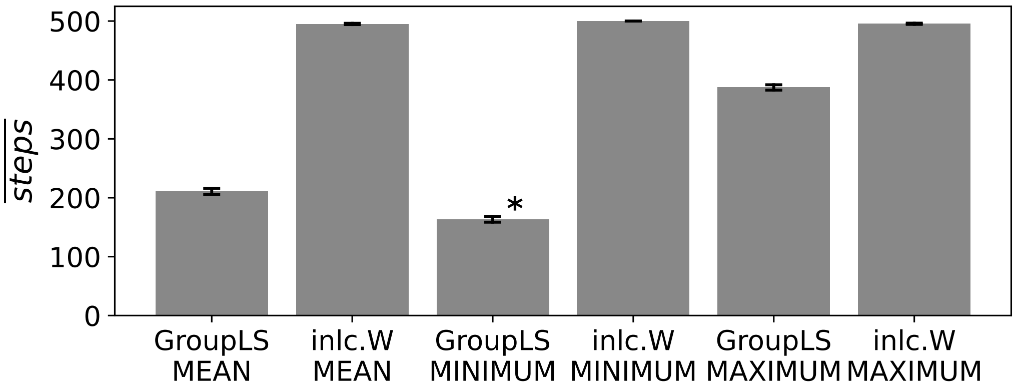

Figure S2. Performance of different reinforcement learning schemes. The average number of steps required for the group of agents to solve the task (y-axis) after 10,000 epochs of training via each reward scheme (x-axis). Fewer steps are better. The * indicates that the MINIMUM reward scheme using group-level selection is performing better than the MEAN group-level selection scheme ($p < 3.89e - 61$; this was the only test performed, as the remaining results are either obvious or—in the case of inclusive fitness—irrelevant). Error bars represent the 95% confidence intervals over 40 samples.

When comparing the performance of individual group members under different training conditions (see Figure S3), it is evident—congruent with the hypothesis—that the MINIMAL reward scheme yields the most equitable distribution. Results from the inclusive fitness group are ignored because learning essentially failed.

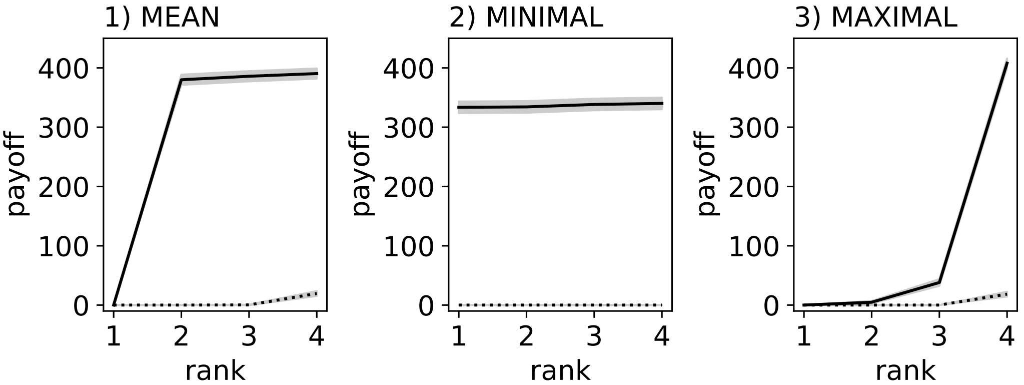

Figure S3. Individual payoff by agents controlled by Q-learning policies. The payoff (y-axis) for the four different agents in the navigation task is sorted in ascending order, evaluated over all combinations of reward schemes. Group-level selection (bold line) and inclusive fitness (dotted line) are shown. Black shadows denote the 95% confidence interval over 40 samples.

This environment was designed to illustrate that different problems—such as this navigation task—can be optimized using the MINIMAL reward scheme. Here, the weakest member is defined as the one that takes the longest to reach its matching outlet. However, this example also demonstrates that care must be taken in designing environments so that they match the specific real-world problem and avoid overly competitive conditions. Hypercompetition makes inclusive fitness selection difficult and prevents the four different policies from converging on a single solution.

2 PARAMETERS OF THE GENETIC ALGORITHMS

Below are the parameters of the genetic algorithm implemented in MABE:

Parameter	Value
Population size	100
Generations	50,000
Start genome length	5,000 sites
Maximal genome length	20,000 sites
Minimal genome length	2,000 sites
Per site mutation rate	0.005
Per genome gene duplication rate	0.1
Duplication size range	128–512
Per genome gene deletion rate	0.1
Deletion size range	128–512
Computational node types	deterministic, probabilistic, mathematical
Selection	asexual, roulette
Deterministic node properties	2–4 inputs, 1–4 outputs
Probabilistic node properties	1–4 inputs, 1–4 outputs
Mathematical node properties	1–4 inputs, 1–4 outputs
Mathematical node operators	0.0, 1.0, constant value, sum, product, sub, divide, ==

Table S1. Parameters of the genetic algorithm used in the experiments.
